# Correction to “Analysis of Ionomer Distribution and Reaction Mechanisms on Polymer Electrolyte Fuel Cell Pt Catalysts Supported on Mesoporous Carbon under Various Humidity Conditions”

**DOI:** 10.1021/acsaem.5c02324

**Published:** 2025-08-21

**Authors:** Kiyotaka Nagamori, Satoshi Aoki, Mayumi Ikegawa, Yasuhiro Seki, Hiroshi Igarashi, Makoto Uchida

In the original paper, in Figure S1 of the Supporting Information, the units in the images must change from μm to nm. Also, the caption of Figure S5 of the Supporting Information must change from “(a) 100% RH, (b) 50% RH, (c) 30% RH” to “(b) 100% RH, (c) 50% RH, (d) 30% RH”.

The corrected Figures S1 and S5 are reproduced in the Supporting Information associated with this Addition and Correction. This error does not affect the results or conclusions of the paper.

## Supplementary Material



## Notes

Same description as in the original paper (PDF)

