# Supplementary material for: Correction to “Analysis of Ionomer Distribution and Reaction Mechanisms on Polymer Electrolyte Fuel Cell Pt Catalysts Supported on Mesoporous Carbon under Various Humidity Conditions”
Source: ACS Appl Energy Mater. 2025 Aug 21;8(17):12996. doi: 10.1021/acsaem.5c02324 (PMC12421496; doi:10.1021/acsaem.5c02324)
Supplement: Supplementary file 1 [file ae5c02324_si_001.pdf]

Supporting Information

Analysis of Ionomer Distribution and Reaction Mechanisms on Polymer Electrolyte Fuel Cell Pt Catalysts Supported on Mesoporous Carbon under Various Humidity Conditions

Kiyotaka Nagamori[1,2], Satoshi Aoki[1], Mayumi Ikegawa[1], Yasuhiro Seki[1], Hiroshi Igarashi[1], Makoto Uchida[3,*]

[1] N.E. Chemcat Corporation, Research and Development Center, Fundamental Research Group, Bando 306-0608, Japan

[2] Interdisciplinary Graduate School of Medicine and Engineering, University of Yamanashi, Kofu 400-8511, Japan

[3] Hydrogen and Fuel Cell Nanomaterials Center, University of Yamanashi, Kofu 400-0021, Japan

* Corresponding author
E-mail address: uchidam@yamanashi.ac.jp (M. Uchida).

doi.org/10.1021/acsaem.5c01411

Table of Contents

Figure S1 TEM images and XRD patterns of the synthesized catalysts S3

Figure S2 Isotherms and their hysteresis volumes at 0.5 P/P_0 in nitrogen adsorption S4

Discussion S1 Additional discussion of measurement of pore volume. S5

Figure S3. Comparison of pore volume about catalyst layer by N_2-PV and Hg-PV. S6

Figure S4. STEM-EDS images of cross-sections of the Pt/MPC catalyst layer S7

Discussion S2 Explanation of onset potential of CO stripping and the effect of measurement conditions S8

Figure S5. Cyclic voltammograms of Pt/MPC catalysts at I/C ratios from 0.7 to 1.3 .... S9

Figure S6. Comparison of ECSA values for different catalysts S10

Figure S7. SEM cross-sectional images and thickness analysis of the cathode catalyst layer........S11

Figure S8. *I-V* curves and Tafel slopes for Pt/K and Pt/MPC S12

Figure S9. HFR-free *I-V* curves and Tafel plots for Pt/MPC catalysts S13

Figure S10. Comparison of gas diffusion resistance components S14

TableS1. Results of Tafel slope step analysis and current density loss for Pt/K S15

Reference S16

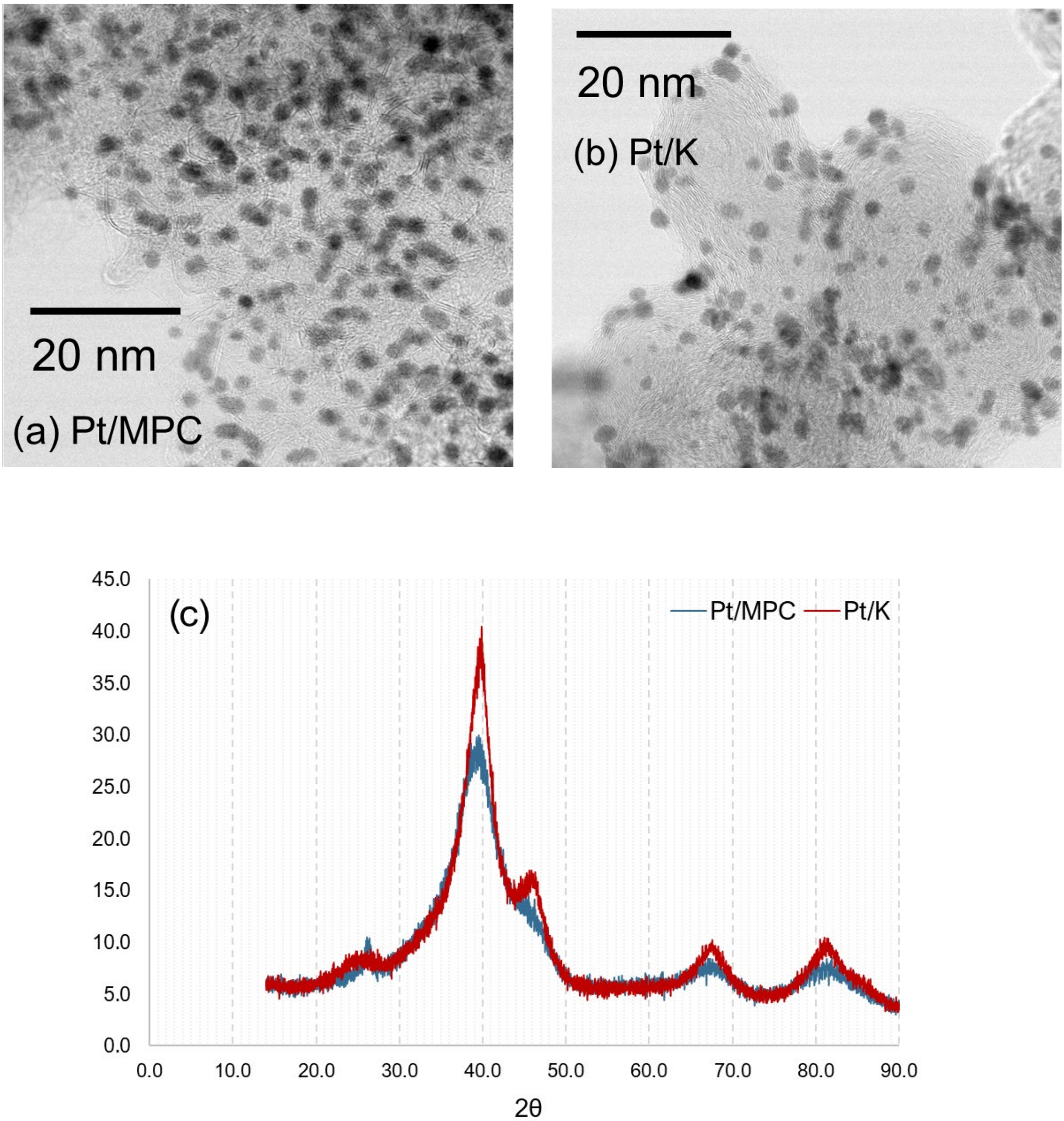

Figure S1. (a) TEM image of Pt/C based on MPC, (b) TEM image of Pt/C based on EC300J, and (c) XRD patterns of each catalyst.

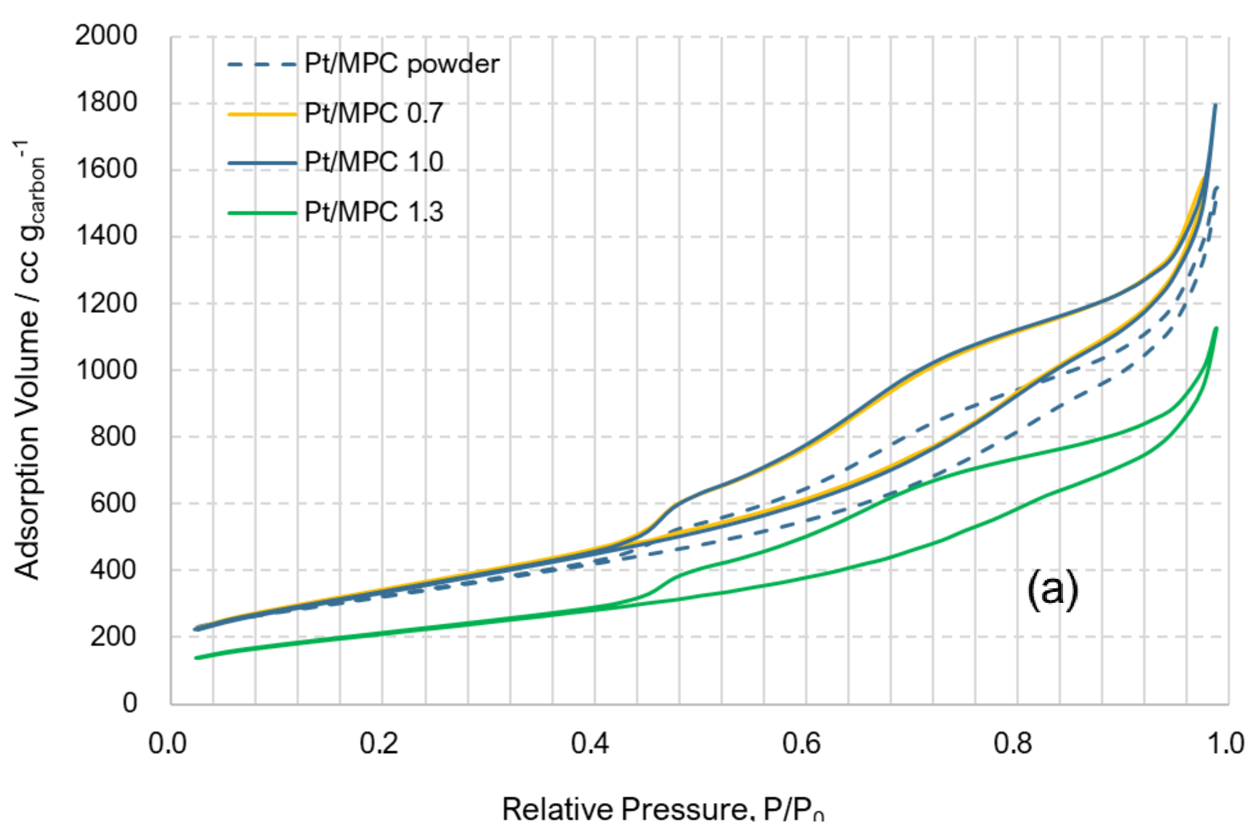

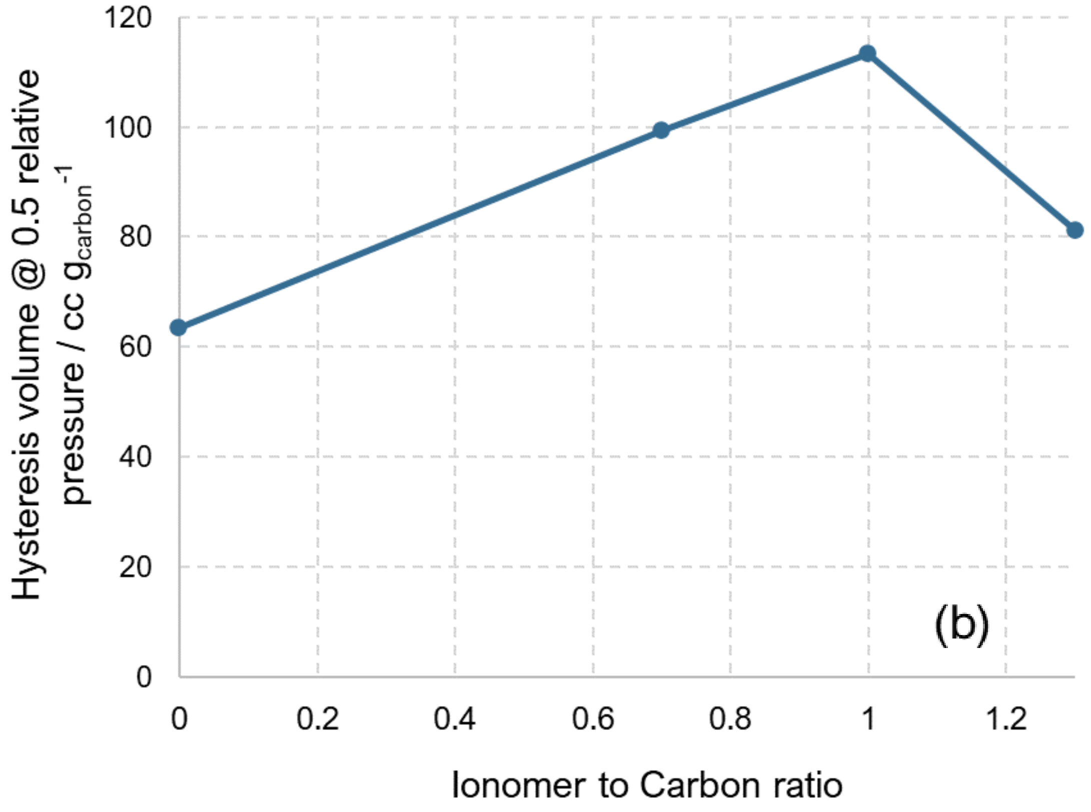

Figure S2. (a) Isotherms and (b) their hysteresis volumes at 0.5 P/P$_0$ in nitrogen adsorption isotherms for Pt/MPC catalysts with varying I/C ratios. I/C ≃ 0 represents the catalyst powder.

Discussion S1. **Additional discussion of measurement of pore volume**

- Comparison of pore volume about catalyst layer by N_2-PV and Hg-PV (Figure S3).

We examined the alterations in pore volume after the incorporation of ionomer into 2 catalysts (Pt/MPC and Pt/K). Although we compared the pore volume using both nitrogen adsorption and mercury porosimetry, the two methods yielded disparate results, as shown in Figure S3. This is attributable to the different principles of pore size determination inherent to each technique. The following provides a supplementary explanation of our findings, which we were unable to elaborate on in the main text.

Since there are large voids between particles in the catalyst layer of Pt/MPC, analyzing the pore distribution in these larger pores is challenging by N_2-PV. This is because at high relative pressures, larger pores may not be filled by the adsorbate. Mercury porosimetry can more precisely represent the occupancy of secondary pore regions by ionomer compared to nitrogen adsorption. However, it is inadequate for pores smaller than 10 nm due to its restricted penetration range. For porous materials like mesoporous catalysts, localized constrictions at the entrances of primary particles render mercury porosimetry less effective for examining small pore regions. The addition of the ionomer greatly enhanced the formation of such narrow pores, an effect that was especially pronounced in the MPC. Consequently, these pore regions are not fully detectable by mercury porosimetry. On the other hand, N_2-PV is well-suited for detecting nanopores(2~10 nm). This highlights the necessity of a complementary approach, utilizing both methods to achieve a comprehensive characterization of the catalyst layer.

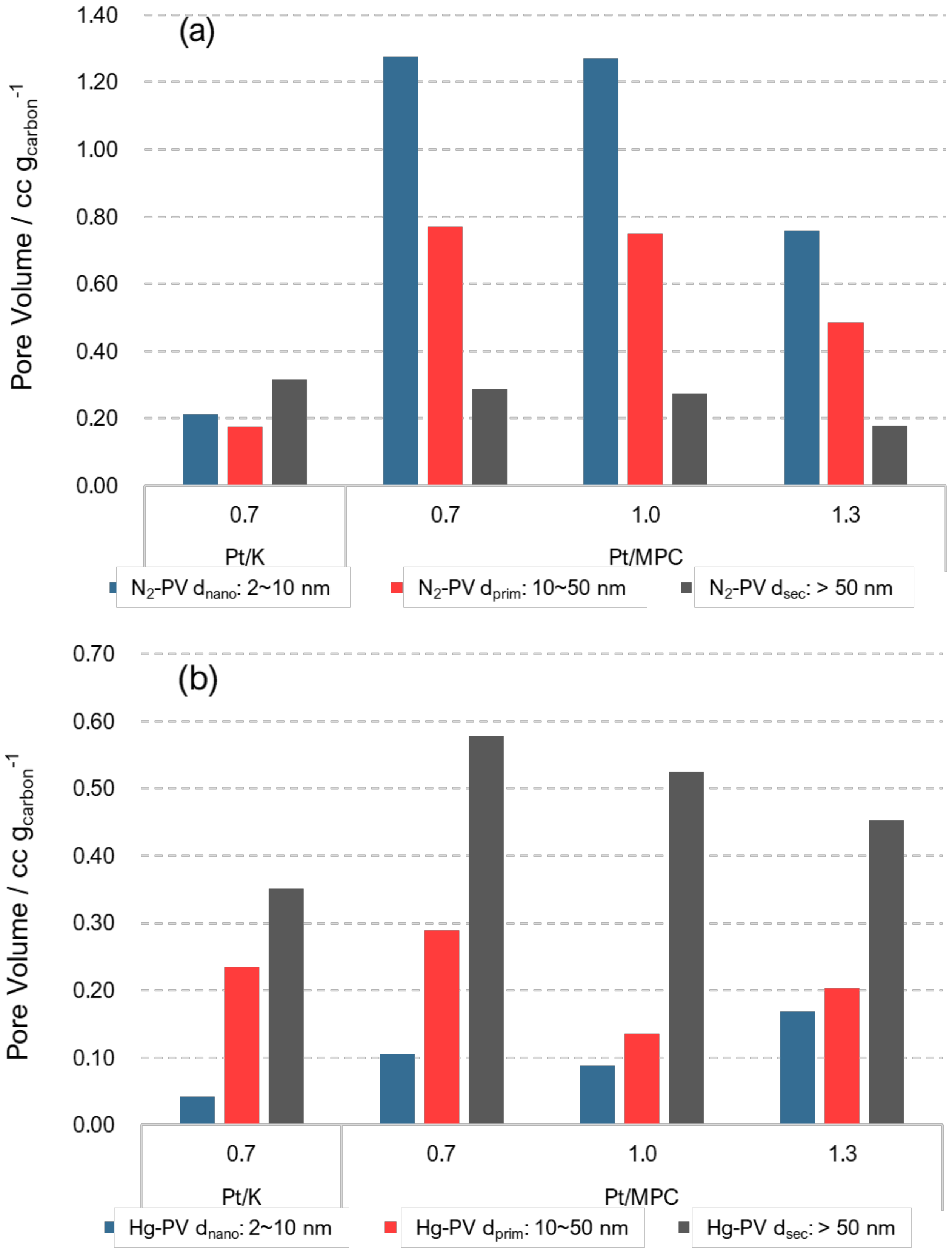

Figure S3. Comparison of pore volume about catalyst layer categorized by pore size definitions: (a) N_2-PV (BJH method) and (b) Hg-PV.

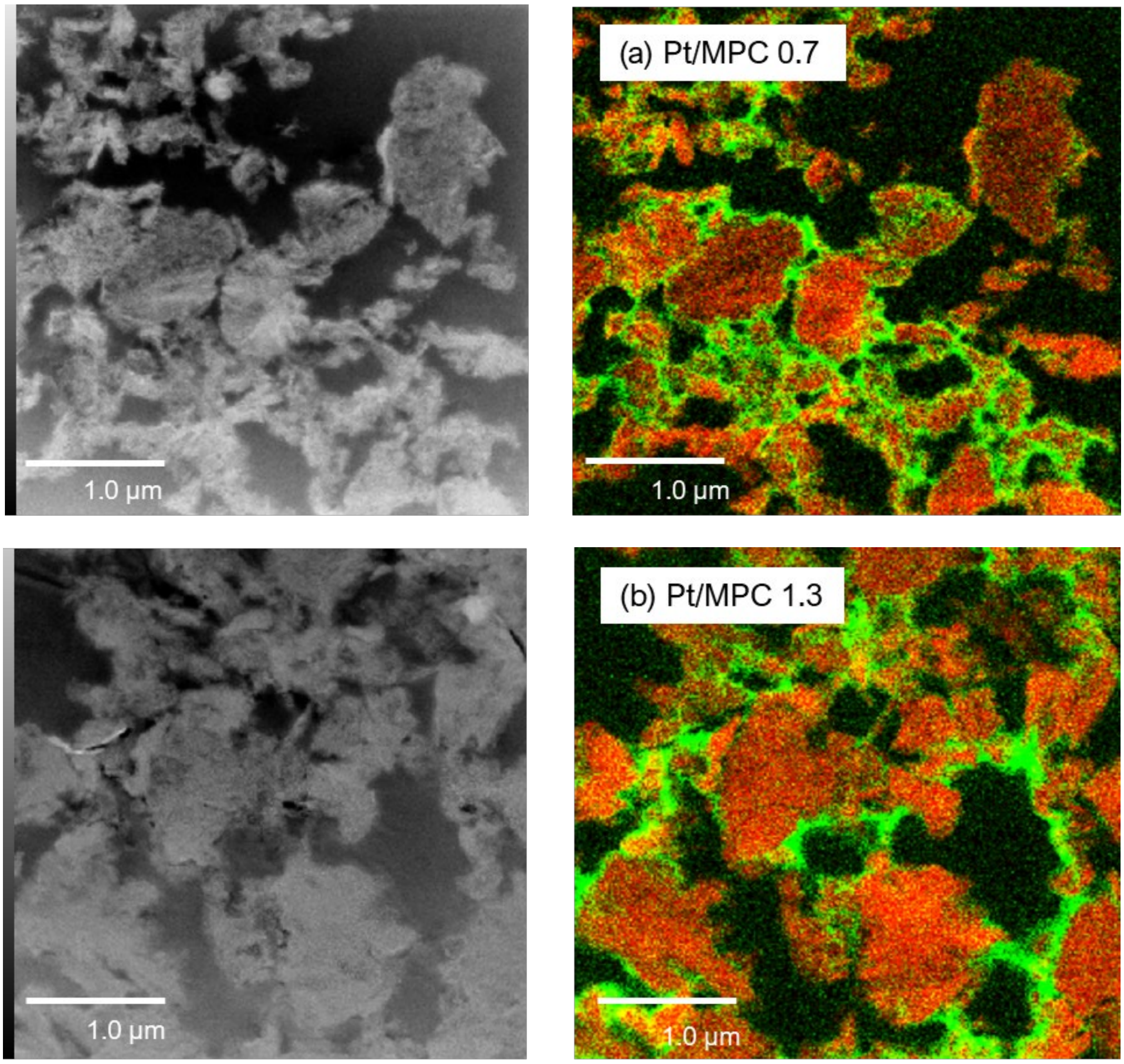

Figure S4. STEM-EDS images of cross-sections of the Pt/MPC catalyst layer (different field of view from the main text). Red represents Pt, and green represents F. (a) I/C 0.7; (b) I/C 1.3.

Discussion S2. **Explanation of CO oxidation onset potential and the effect of measurement conditions**

- CO oxidation onset potential at high and low humidity conditions.

At 100% RH, both the peak position and intensity remained largely unchanged for I/C ratios of 1.0 and 1,3, while a slight delay in CO oxidation onset was observed for I/C 0.7 (FigureS5(b), (c)). A significant anodic shift in the onset potential for CO stripping was observed at 30% RH. The onset potential of CO stripping indicates the initiation of the CO oxidation reaction, and a higher potential suggests that the reaction is more difficult to start (FigureS5(d). We believe this peak shift is consistent with the explanation provided by Shinozaki et al [1]. We assert that this result emphasizes the development of the ionomer network on the outer surface of primary particle as described in the main text.

- Temperature difference between the two methods in ECSA measurement.

In the main text, we discuss the discrepancy between the ECSA values calculated from CO stripping and those from the hydrogen adsorption/desorption (HAD) waves. Here, we provide an additional explanation for the reason why these measurements were conducted under different conditions.

This study involved ECSA measurements at varying temperatures for two methods: CO stripping at 60°C and HAD at 40°C. HAD at nearly 40°C demonstrates an ECSA value approaching its maximum owing to temperature dependence [2]. Although it also has been reported that higher ECSA values can be obtained from CO stripping at lower temperatures, we adopted 60°C for our measurements [3]. Because we sought to prevent the misinterpretation of ECSA due to the CO stripping peak shifting excessively to higher potentials at 40°C, overlapping with the Pt redox peaks. For both measurement methods, we selected temperature conditions that allow for the acquisition of the highest possible ECSA value while minimizing measurement errors.

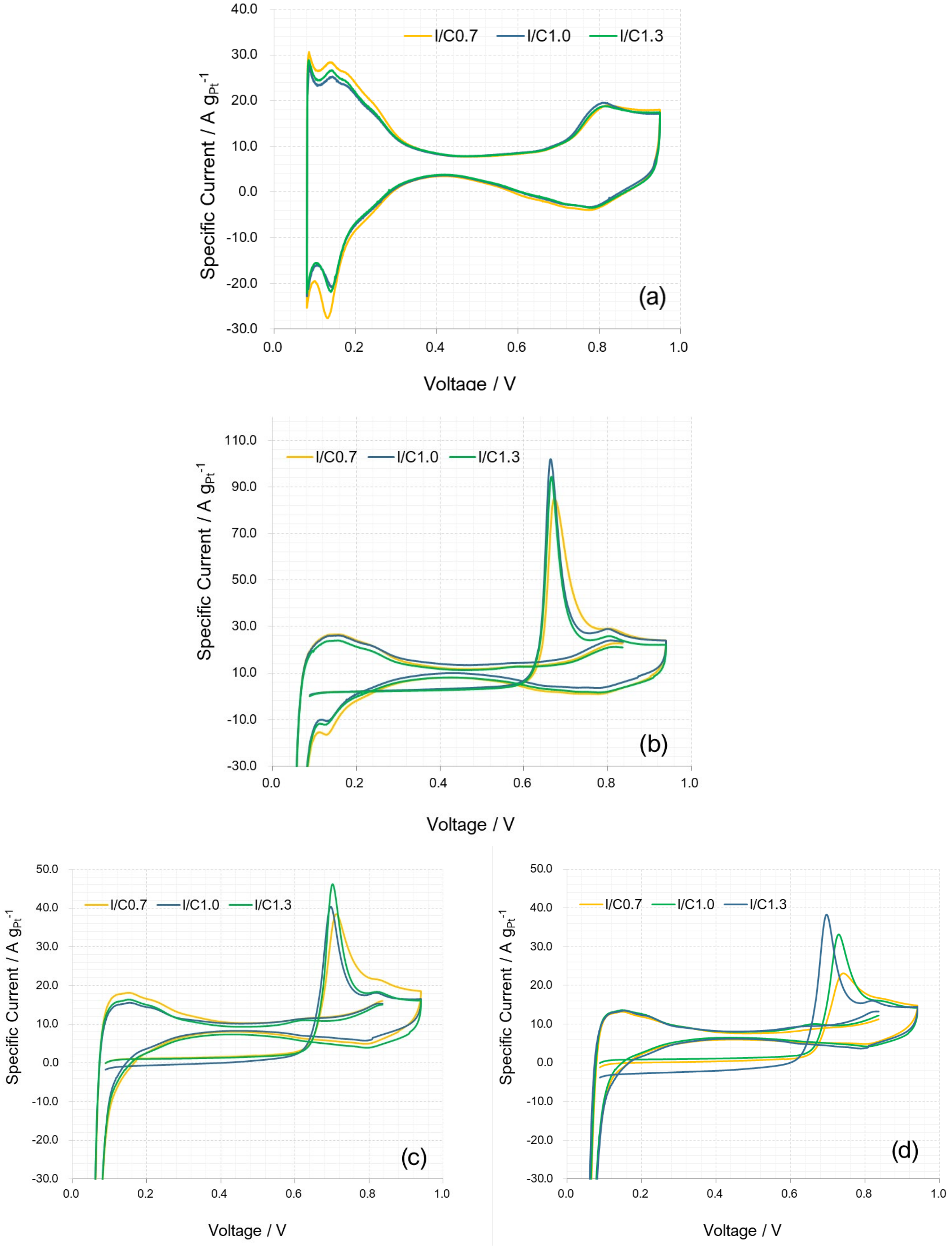

Figure S5. Cyclic voltammograms of Pt/MPC catalysts at I/C ratios from 0.7 to 1.3: (a) hydrogen adsorption/desorption, (b) CO stripping for Pt/MPC catalysts at various I/C ratios and relative humidity conditions: (b) 100% RH, (c) 50% RH, (d) 30% RH. (first cycle and partway through the second cycle).

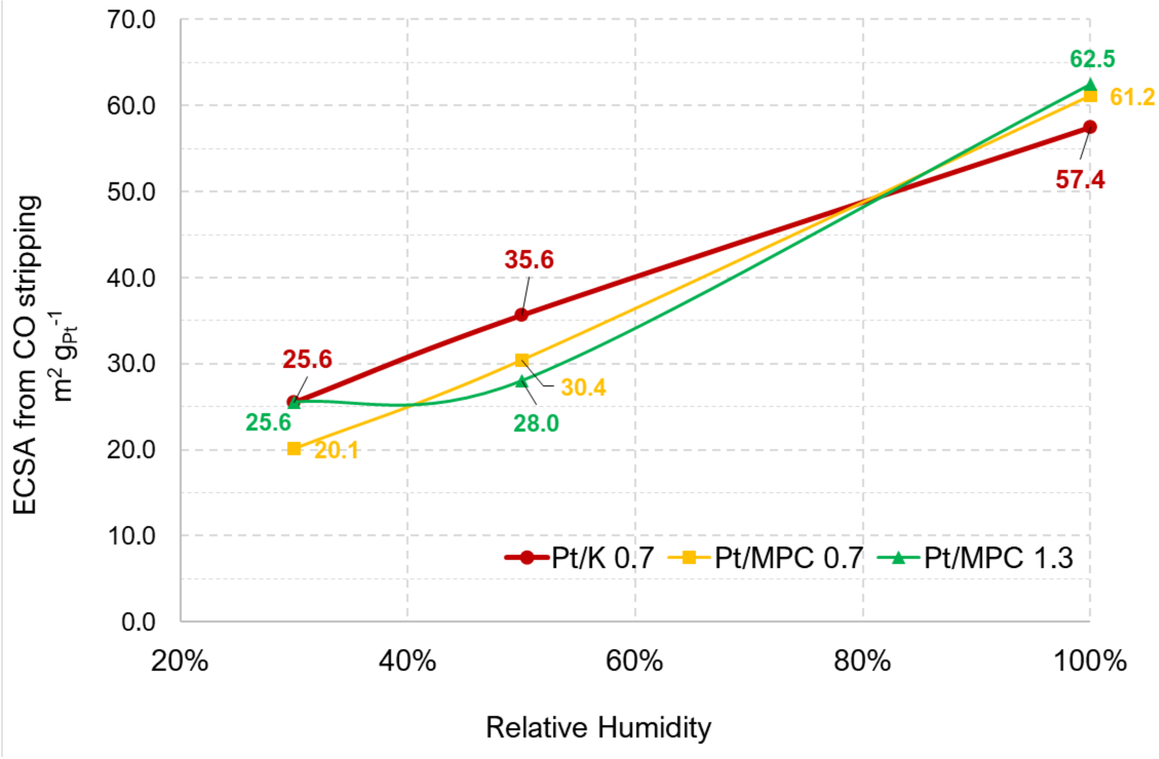

Figure S6. Comparison of ECSA values calculated from CO stripping curves for different catalysts and ionomers under 100%,50%%, and 30% relative humidity conditions (Pt/MPC values are taken from those presented in the main text).

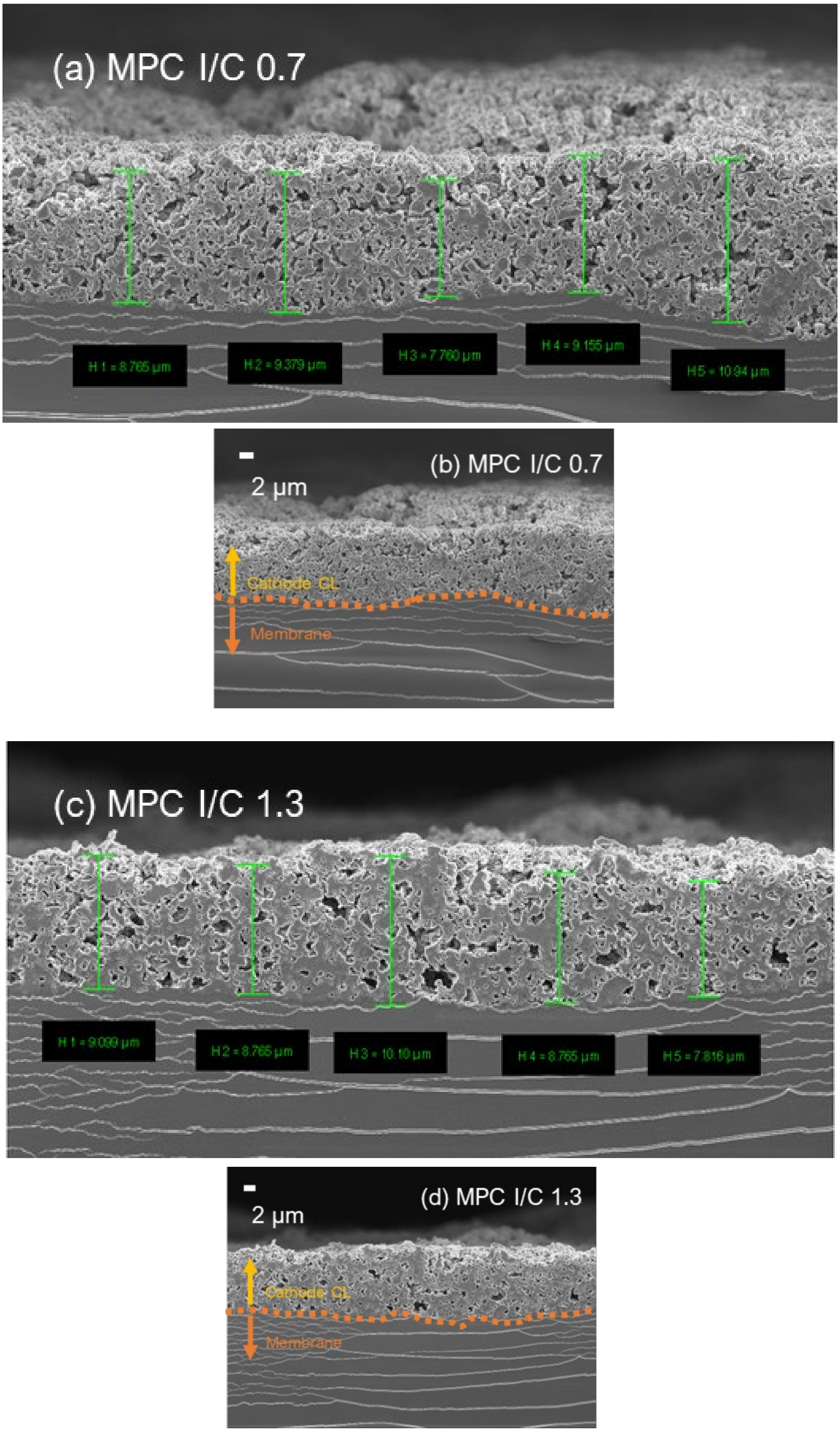

Figure S7. SEM cross-sectional images and thickness analysis of the cathode catalyst layer in Pt/MPC for (a), (b) I/C 0.7 and (c), (d) I/C 1.3.

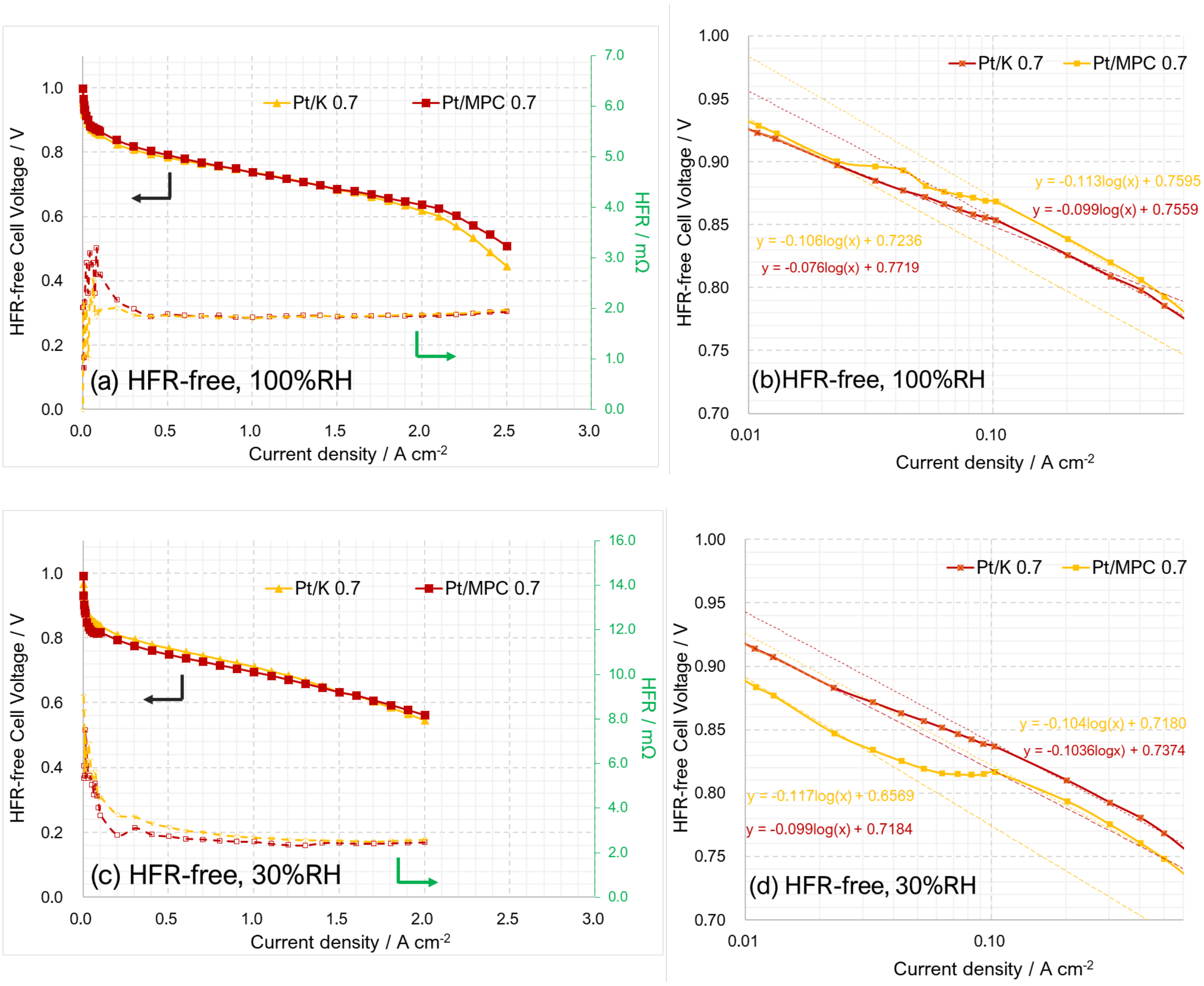

Figure S8. *I-V* curves and Tafel slopes for Pt/K and Pt/MPC at the same I/C ratios: (a), (b) 100% RH; (c), (d) 30% RH. (Data for Pt/MPC are taken from the main text.)

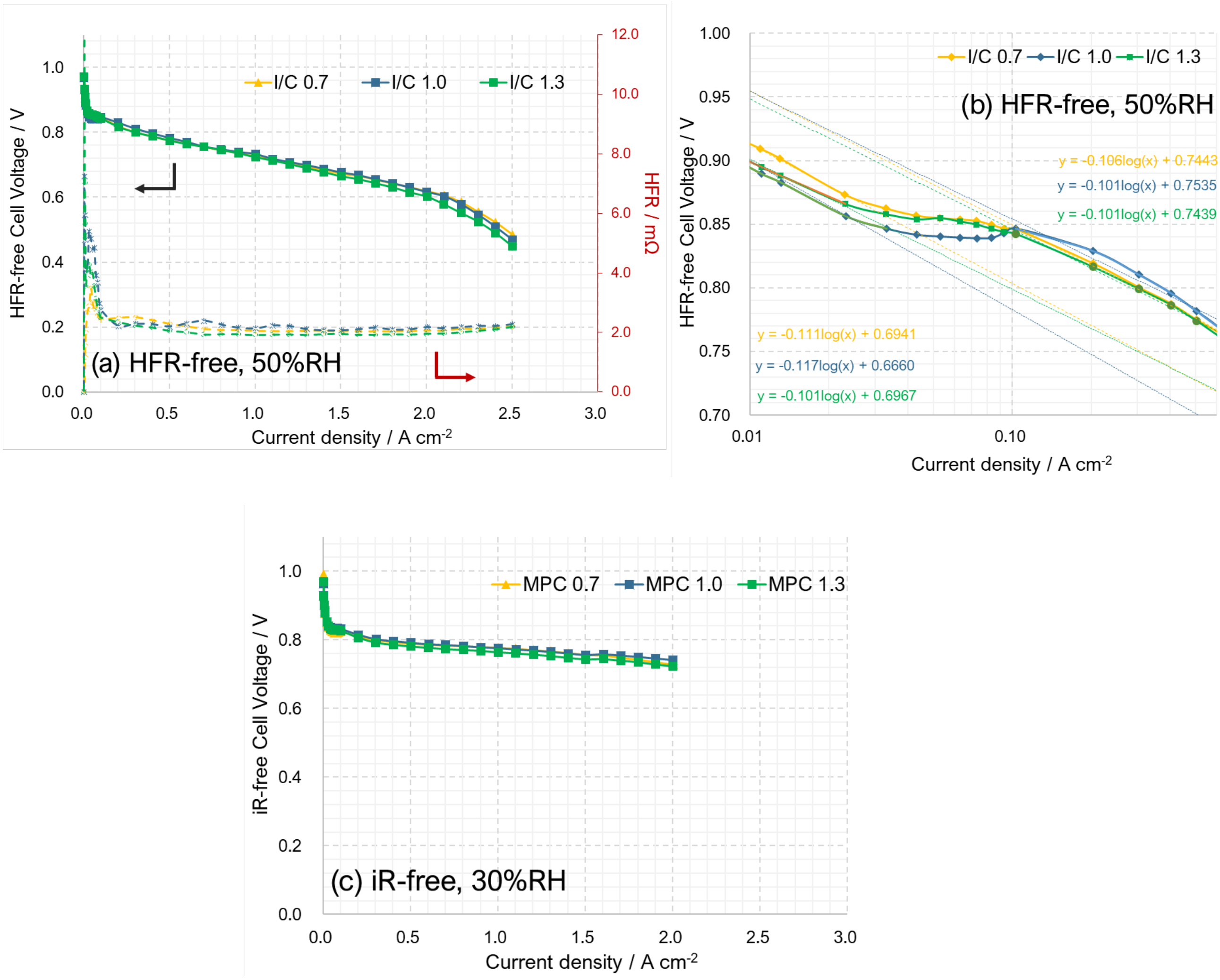

Figure S9. HFR-free *I-V* curves and Tafel plots for Pt/MPC catalysts at various I/C ratios under 50% RH, and *iR*-free *I-V* curves corrected for proton resistance under 30% RH.

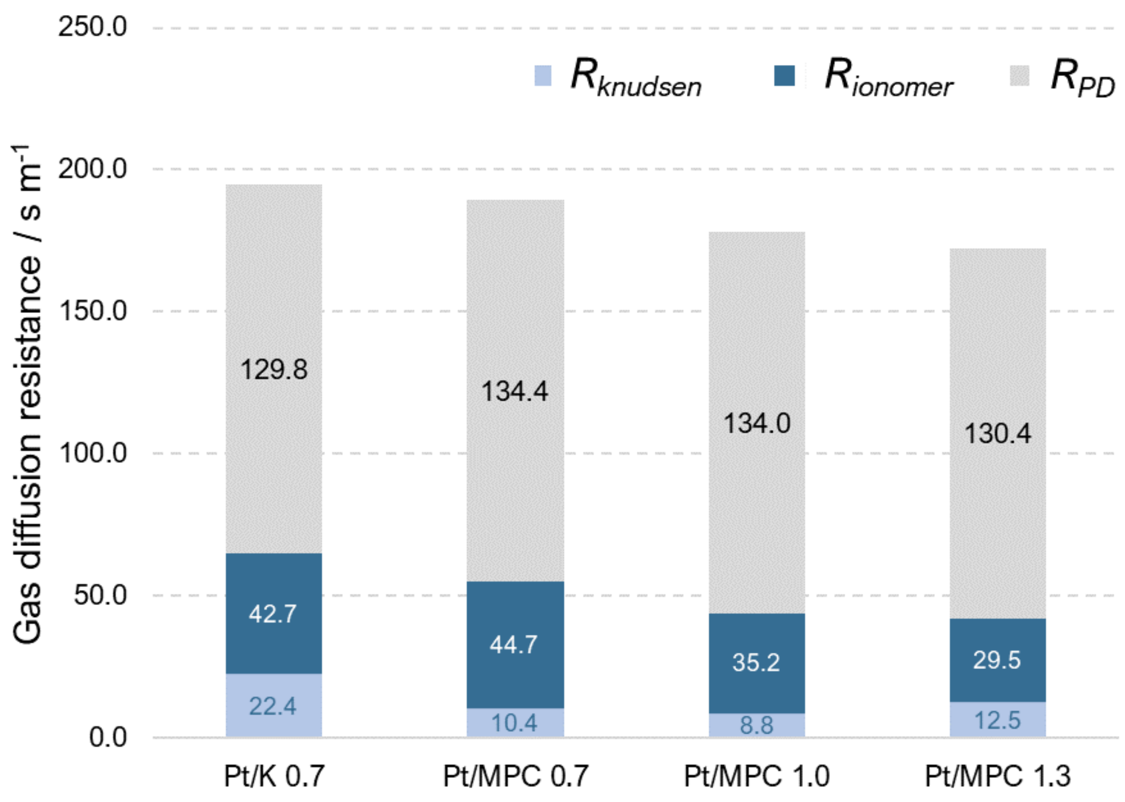

Figure S10. Comparison of gas diffusion resistance components for Pt/K and Pt/MPC with different I/C values (including R_{PD} derived from molecular diffusion, which was not described in the main text).

Table S1. Results of Tafel slope step analysis and current density loss (CD loss) for Pt/K (I/C0.7) at different relative humidity levels.

I/C	Ref @0.1 A cm^{-2} V			Higher CD A cm^{-2}			Lower CD A cm^{-2}			CD Loss		
	100% RH	50% RH	30% RH	100% RH	50% RH	30% RH	100% RH	50% RH	30% RH	100% RH	50% RH	30% RH
Pt/K 0.7	0.854	0.839	0.837	0.103	0.108	0.110	0.085	0.091	0.064	18%	16%	42%

■ Reference

1) Shinozaki, K.; Yamada, H.; Morimoto, Y. Relative Humidity Dependence of Pt Utilization in Polymer Electrolyte Fuel Cell Electrodes: Effects of Electrode Thickness, Ionomer-to-Carbon Ratio, Ionomer Equivalent Weight, and Carbon Support. *J. Electrochem. Soc.* **2011**, *158* (5), B467.

2) Lee, M.; Uchida, M.; Yano, H.; Tryk, D. A.; Uchida, H.; Watanabe, M. New evaluation method for the effectiveness of platinum/carbon electrocatalysts under operating conditions. **2010**, *Electrochim. Acta*, *55*(28), 8504–8512.

3) Garrick, T. R.; Moylan, T. E.; Carpenter, M. K.; Kongkanand, A. Editors' Choice—Electrochemically Active Surface Area Measurement of Aged Pt Alloy Catalysts in PEM Fuel Cells by CO Stripping. *J. Electrochem. Soc.* **2017**, *164* (2), F55–F59.
